# Ageing and Quality of Life in Older Adults: Updates and Perspectives of Psychosocial and Advanced Technological Interventions

**DOI:** 10.3390/healthcare14020217

**Published:** 2026-01-15

**Authors:** Dinara Sukenova, Dejan Nikolic, Aigulsum Izekenova, Ardak Nurbakyt, Assel Izekenova, Jurate Macijauskiene

**Affiliations:** 1 Department of Public Health, Asfendiyarov Kazakh National Medical University, Almaty 050012, Kazakhstan; sukenova.d@kaznmu.kz (D.S.); a.nurbakyt@kaznmu.kz (A.N.); 2 Faculty of Medicine, Kenzhegali Sagadiyev University of International Business, Almaty 050012, Kazakhstan; 3 Department of Physical Medicine and Rehabilitation, University Children’s Hospital, 11000 Belgrade, Serbia; 4 Faculty of Medicine, University of Belgrade, 11000 Belgrade, Serbia; 5 Department of Epidemiology with the Course of HIV, Asfendiyarov Kazakh National Medical University, Almaty 050012, Kazakhstan; izekenova.a@kaznmu.kz; 6 Center for Social and Business Research, Kenzhegali Sagadiyev University of International Business, Almaty 050012, Kazakhstan; izekenova.a@uib.kz; 7 School of Social Work, Michigan State University, East Lansing, MI 48824, USA; 8 Department of Geriatrics, Lithuanian University of Health Sciences, 44307 Kaunas, Lithuania; jurate.macijauskiene@lsmu.lt

**Keywords:** ageing, older adults, multimorbidity, psychosocial interventions, advanced technology, quality of life

## Abstract

Expanding longevity, together with a decrease in mortality, leads to an increase in the older population worldwide. In this review, ageing and older adults, as well as psychosocial and advanced technological interventions, will be discussed. Older adults are associated with an increased incidence of multimorbidity and disability; thus, they have a higher demand for health services than younger individuals. Challenges in welfare services and inadequate family and community-based care support negatively impact the psychosocial and economic wellbeing of older people. Active ageing and successful ageing are crucial aspects for a better quality of life in this age group, as there is a complex interplay of different domains and disease types that influence quality of life in older adults. Additionally, promoting the social participation of older adults is vital for improving their quality of life. Furthermore, the use of technology in older adults has a positive impact on their quality of life; however, aside from the promotion and implementation of technological interventions, challenges persist at all levels of acceptance and use. A better understanding of these challenges and implementing measures to overcome them will have a significant impact on the technological acceptance of older adults and their use in daily life activities, resulting in more favourable quality of life outcomes.

## 1. Introduction

An increase in longevity, as well as a decrease in mortality, leads to an increase in the older population worldwide [1]. This brings the preservation of functional ability, psychosocial wellbeing, as well as quality of life of the older population into focus of clinical as well as public health investigations and interventions.

According to the systematic analysis for the Global Burden of Disease 2019 study, the global population is experiencing additional years of life; therefore, the health and wellbeing of older adults is important to maintain active engagement in society [2]. In the World Health Organization (WHO) report on Ageing and Health, it was noted that the proportion of the population aged over 60 years worldwide will nearly double from 12% to 22% between 2015 and 2050 [3]. Furthermore, by 2030, the proportion of the population 60 years of age and above will increase to 1.4 billion from 1 billion in 2020, meaning one in six people worldwide will be aged 60 years and above [3].

Changes in the proportion of older adults are influenced by the birthrate and survival of the one [4]. In Europe, it is projected that by 2030, one in four Europeans will be 65 years of age and above [4]. In Asia and the Pacific, one in four people will be aged 60 years and above by 2050, reaching the proportion of nearly 1.3 billion people [5]. According to the Population Reference Bureau, the proportion of Americans aged 65 years and above is projected to rise from 17% to 23% between 2022 and 2050 [6].

In developed countries, the majority of older people live in areas that are classified as urban, while in developing countries, such individuals live in rural areas and in multigenerational households [7]. The oldest age (individuals 80 years and above) is the fastest-growing segment of the older population [7].

## 2. Rationale and Objectives

Psychosocial interventions are shown to have potential for improvement of the quality of life in older adults, bearing in mind the importance of their integration into multidisciplinary models of care. Recognizing their optimal and full impact on the quality of life in this age group is vital. Therefore, a better understanding of the present challenges in terms of barriers and facilitators, as well as gaps and motivations for psychosocial interventions implementation in a continuously changing society, is needed and justified.

Research on technology in the context of ageing has grown significantly in recent years. According to Pruchno, high costs of care for older adults with disabilities, as well as rapid technological advances in healthcare, are reported to be responsible for such growth [8]. The introduction and implementation of advanced technologies, particularly during the COVID-19 pandemic, in older adults found its place that was influenced more by the need than the readiness. The vital role of technology in addressing the challenges of an ageing population is reflected in the fact that technological applications provide support for ability declines, increase engagement opportunities and safety, and facilitate access to services [9]. In the post-pandemic period, it is important to propose and implement technologies that are person-oriented, acceptable, and easy to adopt and navigate in the ageing care. However, the need to identify and address the potential gaps and motivations regarding technological use in older adults is of significant importance.

Considering psychosocial interventions, the objectives of this review are oriented toward the evaluation of the role and impact of these interventions on the improvement of multidimensional aspects of the quality of life in older adults.

The additional objectives are directed toward the quality-of-life-oriented aspects concerning technological interventions in older adults. Despite the advantages of the technological applications in older adults, challenges remain, particularly in terms of the digital divide, inadequate integration and adaptation, leading to further barriers that are ultimately impacting their quality of life. Therefore, the importance of motivations for technology use and contemporary approaches for a better understanding of the interactive balance of acceptance and use of technology in older adults is vital for navigating the positive technological adoption trends in this population group. The potential prerequisites for such favourable outcomes are a better understanding of certain aspects and characteristics of an ageing population, bearing in mind their bio-psycho-social determinants, as well as ageing with its core components that include active, successful, and cognitive determinants, which will be discussed as well in this review.

For this study, further keywords were included: older adults, older people, elderly, age, ageing, multimorbidity, health service, welfare service, institutionalisation, COVID-19, psychological interventions, social interventions, psychosocial interventions, technology, robots, artificial intelligence, smart homes, and quality of life.

The searching database of representative literature included PubMed and additional grey literature.

## 3. Ageing, Older Adults, and Multimorbidity

In the systematic review of the literature by Marengoni et al., published in 2011, it was stated that infectious diseases, during the last century, have been replaced by chronic health problems as the dominant healthcare burden, where almost all chronic conditions are strongly associated with ageing [10]. This aligns with the fact that chronic dysregulation of multiple organ systems is associated with ageing [11]. Moreover, functional impairment, depression, distress, poor quality of life, as well as high healthcare utilisation and costs are consequences of multimorbidity [10]. Furthermore, in the editorial by Drazen and Fabbri, published in 2014, it was reported that an increasing proportion of older adults have multimorbidity, which is associated with institutionalisation, disability, poor quality of life, increased rate of treatment adverse effects, and death [12]. There is a negative association between multimorbidity and health-related quality of life, with evidence indicating that the physical health domain is more affected than the mental health domain [11]. Moreover, it was suggested that the association of morbidity with mortality could be mediated by disability [11].

It is reported that the prevalence of multimorbidity in adults aged 60 years and over is between 55% and 98% [13], while in individuals aged 85 years and above, it is probably greater than 80% [14]. Additionally, in a systematic review by Nicholson et al., published in 2024, it was stated that there is a growing concern about the inappropriately high number of medications used in older adults with multimorbidity, where the expected benefit is exceeded by cumulative harm [15]. Furthermore, the authors pointed out that the aim of adequate polypharmacy is to increase longevity, reduce harm, support adherence, and maintain quality of life [15].

In a hospital-based retrospective cohort study by Yen et al., conducted on 995 patients from Taiwan, where data was retrieved from 2018 to 2019, it was reported that for patients with multimorbidity, there is care fragmentation along with suboptimal quality of care in medication appropriateness and patient preference, as well as goal setting based on recommendations from disease-specific guidelines [16]. Moreover, these authors stated that there is a positive impact of integrated outpatient services for older adults with multimorbidity on quality of life [16]. Additionally, in a propensity score-matched cohort study on older patients with multimorbidity by Lo et al., conducted in Taiwan on 166 patients included in integrated ambulatory care programme (IACP) and 664 non-exposed patients between 1 June and 31 December 2019, the authors stated that patients with multimorbidity may benefit from expanding the integrated ambulatory care programmes in reducing care fragmentation, reducing six-months follow-up at outpatient clinics, as well as spromoting sustainability of the healthcare system [17].

The presence of multimorbidity and its complex interactions with other aspects of older people’s lives present a contemporary challenge not just to the medical approach in achieving optimal treatment results but to society as well, leading to the necessity of proposing specific policies and introducing interventions for overall benefits, better treatment outcomes, and social inclusion and participation.

## 4. Older Adults, Health/Welfare Services, and Institutionalisation

It has previously been noted that older adults are considered to be the most vulnerable group to disease and disability; thus, this age group has a higher demand for health services than younger individuals [18]. As previously stated, older adults have a higher occurrence of multimorbidity, which is associated with a higher frequency and higher costs of health service use [19]. In a systematic review and meta-analysis by Rodrigues et al., published in 2022, it was reported that higher hospitalisation risk and higher hospital readmission rates in older adults are associated with multimorbidity [20]. Furthermore, in a systematic scoping review of provision of health services for older adults in rural and remote areas in Australia, published in 2023, it was stated that access to health services for older populations is a complex issue, with a need to promote positive experiences for both patients and health providers for healthy ageing of those living in rural and remote areas [21].

Previously, the literature reported numerous predictors of the institutionalisation process of the older adults, including advanced age, being without a partner or home, poor self-related health status, lack of assistance in daily living, low educational level, polypharmacy, as well as functional and cognitive impairments [1,22]. In a systematic review and meta-analysis by de Medeiros et al., published in 2020, it was stated that institutionalisation of older adults is associated with poor quality of life [1]. Moreover, in a systematic scoping review by Fealy et al., published in 2024, the authors reported that transitioning into residential age care for older adults is associated with psychological distress for many, with symptoms such as depression, confusion, anxiety, loss, and loneliness [23]. This clearly indicates that preventive and more affirmative measures should be proposed and implemented, bearing in mind all domains of quality of life, including health, phycological and social.

In the era of mobile and wireless technologies expansion with their implementation in the healthcare sector, the e-health, also known as the m-health field, is emerging [24]. The advantages of m-health, particularly for older adults, could be effective disease management as well as an expansion of quality of life [24]. Previous reports stressed that m-health could be used for disease prevention, chronic disease management, and lifestyle modification support in the older population [25].

Finally, older adults experience challenges in welfare services as well as a lack of family and community-based care supports [26]. These challenges negatively impact the psycho-social and economic wellbeing of these individuals [26].

In 2012, the European Innovation Partnership on Active and Healthy Ageing (EIP on AHA) was launched within the Innovation Union policy of the European Commission (EC). Within such an initiative, coordinated activities between Reference Sites (RSs) and Action Groups (AGs) result in the creation, development, and dissemination of innovative solutions for Active and Healthy Ageing [27]. However, one of the main challenges that innovative services face in terms of adoption is cultural resistance; thus, the evolution of the AGs and close engagement of RSs is important in overcoming this barrier [27]. Such an initiative, after eight years in operation, demonstrated that interactions between AGs and RSs have positive effects in transferring innovations [27]. Ultimately, the obtained knowledge and gained experience within this initiative will have positive effects on future developmental and implementation action initiatives on digital health and health systems transformation.

## 5. Bio-Psycho-Social Determinants of Ageing

Ageing is considered to be a dynamic and irreversible physiological process ongoing over time that occurs in biological, psychological, and social spheres [28].

Biological ageing can be described as irreversible changes occurring naturally that increase with age in metabolism as well as the physicochemical characteristics of cells, resulting in structural and functional changes in tissues and organs [28]. Furthermore, it is stated that biological ageing is a complex process, and it is assumed that it is influenced by multiple dysregulated cellular and biochemical processes [29]. Mechanisms of biological ageing are interdependent and can be modified by therapeutic interventions [30].

Social ageing can be considered as the ageing processes and outcomes which are formed by societal factors in their meaning and experiences, and by the ageing social construction [30]. Considering social and emotional functioning, it was pointed out that over the life course, the devastating consequences of isolation do not diminish, nor does the need to feel belonging to a large social group, keeping in mind that strong and intense emotions remain [31]. It should be stated that cognitive functions can be influenced by social spheres in old age, where older adults with increased levels of social activity, as well as strong social networks, have a lower risk of cognitive functioning decline compared to counterparts with opposite social activities [31]. Furthermore, positive emotions in social interactions are reported to be of particular importance for cognitive functioning in social interactions [31].

When addressing psychological ageing, psychological wellbeing is an important factor to be considered. In addition to this, three different aspects of psychological wellbeing have previously been evaluated, including life evaluation, hedonic wellbeing and eudemonic wellbeing [32]. Furthermore, increased risk of having physical illness is associated with impaired wellbeing [32]. In a systematic review by Kang and Kim, published in 2022, it was noticed that older adults with high levels of psychological wellbeing, including having fewer negative emotions, being proud of their age group, a higher optimistic state regarding ageing and the future, being flexible in setting goals, as well as more self-confident regarding their bodies, might be less negatively affected by ageism [33]. In a psychological theory of ageing by Wernher and Lipsky, published in 2015, it was argued that according to a variety of theories, older adults seem or are actually happier than their younger counterparts, which can be explained by the assumption that regulation of conflict and emotions is better in older adults than in younger counterparts [34]. Moreover, these authors stated that the role of cognitive control is an important factor in emotional wellbeing in later life [34].

Finally, the importance of age stereotypes is discussed in the study by Levy, published in 2009, where it was proposed that age stereotypes appear to exert their influence along three pathways: psychological, behavioural, and physiological [35]. Furthermore, in this study, it was argued that expectations are an example of the psychological pathway, health practices of the behavioural pathway, and the autonomic nervous system as an example of the physiological pathway [35].

## 6. Active Ageing, Successful Ageing, and Cognitive Ageing

**Figure 1:**
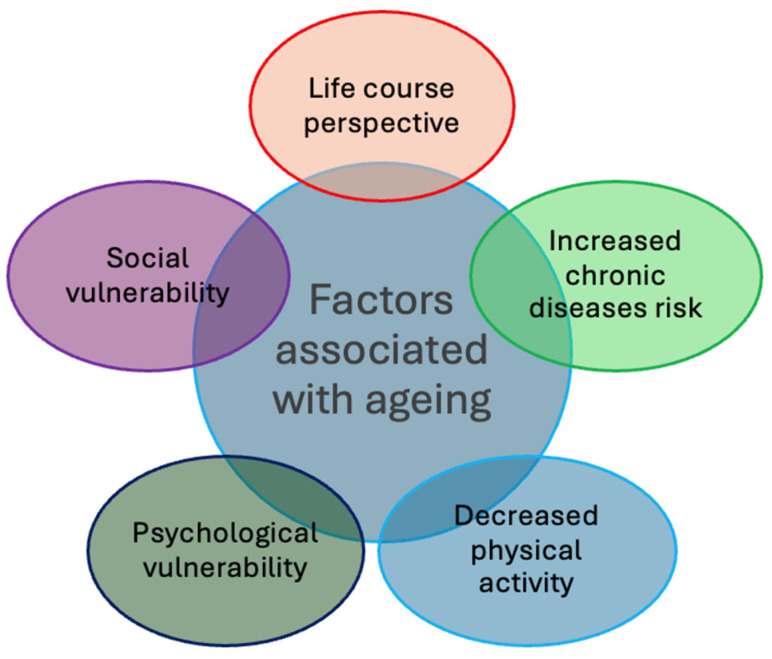
Factors associated with ageing.

Active ageing is the term that was adopted by the WHO in the late 1990s and refers to the process of optimising opportunities for health, participation, and security for the purpose of enhancing quality of life as people age [36]. Furthermore, active ageing enables individuals to recognise their potential for physical, mental, and social wellbeing [36]. Ageing is associated with numerous individual and collective challenges, with a higher proportion of people at risk of chronic diseases, as well as decreased physical activity, psychological, and social vulnerability [37]. Furthermore, it has previously been reported that the process of ageing is strongly shaped by the life course perspective, implying that ageing is biologically and socially constructed [38]. Factors associated with ageing are presented in Figure 1 that is the original illustration created by the authors for this review.

Aside from active ageing, successful ageing is also an important aspect of the ageing process. The model of successful ageing proposed by Rowe and Kahn, published in 1997, includes three main domains: avoidance of disease and disability, preserving high cognitive and physical functioning, as well as active engagement with social and productive activities [38]. In a study on a successful ageing in Canada on a population-based sample of older adults, conducted between 2008 and 2009, where data was from the Canadian Community Health Survey: Health Ageing (CCHS-HA), it was pointed out that being younger, married, perceived better health, those who regularly drink and exercise, as well as those who are satisfied with life and those who were taking calcium in the past month, were associated with successful ageing [39]. Moreover, in the study by Jang et al., on a community-representative sample of non-institutionalised older adults residing in two cities in Korea, conducted between February and March 2003 on 1825 individuals aged 65 years and above, those with successful ageing were more likely to have higher personal income and higher education [40].

In the study by Harada et al., published in 2013, it was stated that a decline in certain cognitive aspects, including processing speed and certain memory, language, visuospatial, and executive function abilities, is associated with normal ageing [41]. These authors also stressed that cognitive reserve building and cognitive retraining engagement might be a path for achieving successful cognitive ageing [41]. Furthermore, Depp et al., in their study from 2010, pointed out that cognitive and emotional health improvement in older adults could be achieved by caloric restriction, physical activity, cognitive intervention, as well as stress reduction [42]. Furthermore, they also argued that there are numerous determinants of successful cognitive ageing, including genetics, stress and resilience, where they pointed out that older adults have different stressors than younger counterparts, as well as the fact that there are diverse responses to stressors in older adults in situations where the nature and type of stressor is similar across people, than brain reserve and cognitive reserve, wisdom and lifestyle behaviours including physical activity, nutrition and dietary restrictions as well as cognitive stimulation [42].

**Figure 2:**
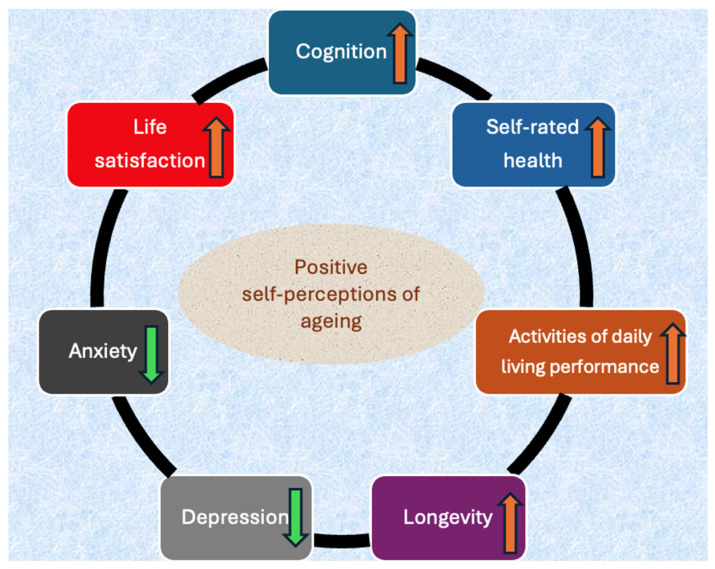
Factors affecting self-perceptions of ageing.

Additionally, better mental and physical health, as well as better satisfaction with life and better cognitive function, were shown to be associated with lower subjective age [43]. In a systematic review by Fernández-Ballbé et al., published in 2023, it was reported that better cognition and lower cognitive decline were associated with positive aspects of self-perception of ageing and a younger subjective age [44]. Moreover, in another systematic review of longitudinal studies by Tully-Wilson et al., published in 2021, it was stated that better self-rated health, performance of activities of daily living and cognitive functioning, as well as increased longevity and lower depression and obesity, were associated with positive self-perceptions of ageing [45]. Furthermore, lower anxiety and higher life satisfaction were shown to be positively associated with self-perceptions of ageing in older adults [46]. In Figure 2 (original illustration created by the authors for this review), factors affecting self-perceptions of ageing are presented.

## 7. Older Population and COVID-19

The COVID-19 pandemic affected older adults in numerous ways. The COVID-19 pandemic transformed the lives of individuals and societies, influencing public policies, including active ageing policy, and threatening all three dimensions of health, dignity, and participation [47]. Numerous challenges associated with the COVID-19 pandemic were reported; among them, in the health sector, was the fact that the coronavirus was a new disease, with a lack of information regarding the treatment, as well as movement restrictions, reduced face-to-face meetings and hospital visits, and financial limitations [48]. In line with this, there is justification for the implementation of adaptive models of health care delivery. These models, including telehealth consultations, have been rapidly adopted for the purpose of securing ongoing delivery of essential health care services [49].

In a scoping review by Mushtaq and Khan, published in 2024, it was reported that during COVID-19, older adults experienced a serious burden of social isolation as well as adverse mental health effects [50]. Furthermore, in a study on 2207 community-dwelling older Canadians regarding quality of life and wellbeing during the COVID-19 pandemic, published in 2023, it was stated that those with multiple chronic conditions and older adults with limited resources could be at increased risk of having adverse quality of life and wellbeing consequences [51]. A qualitative systematic literature review of older adults’ experiences during the COVID-19 pandemic, published in 2023, highlighted the impact of the pandemic on social connectivity and wellbeing of older adults in terms of the absence of loved ones’ proximity, resulting in increased anxiety, depression, and loneliness [52]. Additionally, it was reported that numerous sociodemographic and health-related factors were shown to be associated with anxiety, loneliness, and depression in older adults during the COVID-19 pandemic [53]. However, adaptation to lifestyle changes and social distancing of older adults was also reported [52].

It was noticed that, aside significant shift of healthcare into the digital world, such a shift extends beyond healthcare into the digital realm, with online access to COVID-19 related news, group socialisation, education and delivery services, with a paradox where the older adults that are mostly negatively affected by the COVID-19 pandemic are being least likely effective in accepting the offered advantages [54]. Therefore, programmes and interventions directed towards adequate and successful inclusion of older adults into the digital future are needed.

## 8. Quality of Life Among Older Adults

Quality of life can be considered a meaningful measure for the assessment of patient-reported outcomes and health care services, providing important information regarding patients’ satisfaction with a focus on organisation, accessibility, and quality of care [13]. In a study by Adhikari et al., conducted in Nepal, between April and May 2023, on 366 older adults, it was noted that numerous factors, such as physical, social, functional, and emotional, which contribute to the health of the individual, are associated with one’s quality of life [55]. Additionally, these authors stated that comorbidities, physical and social activities, education, income, healthcare affordability, and access to health services are factors associated with quality of life [55]. In a systematic review by Marzo et al., published in 2023, it was reported that several domains of quality of life in older adults were consistently and positively associated with active ageing, where social participation, reading, art, physical activities, financial security, and ensuring care had a positive impact on quality of life [56]. It was reported that an increase in the level of health literacy increased levels of health empowerment and quality of life, while an increase in health empowerment levels increased the level of quality of life in individuals aged 65 years and above [57]. Moreover, in the systematic review by Velaithan et al., published in 2024, it was stated that increased quality of life was associated with a positive perception of ageing, and that more positive perceptions of health were associated with higher morale and good physical capability in older adults [46]. This implies that quality of life in older adults can be considered multidimensional, with different roles for numerous domains.

In the WHO report on healthy ageing and functional ability, healthy ageing is defined as “the process of developing and maintaining the functional ability that enables wellbeing in older age”, while functional ability is described as the capabilities which enable individuals to be and do what they have reason to value, including meeting basic needs, learning, growing and making decisions, mobility, building and maintaining relationships and contribution to society [58].

Regarding health-related quality of life, in a cross-sectional study on 8786 home-dwelling older adults 75 years and older from Switzerland, conducted in 2019, it was pointed out that better socio-economic status in terms of better income and having supplementary insurance, along with a higher level of education, were positively associated with health-related quality of life [59]. Furthermore, in a multivariable analysis by Geigl et al., conducted on 1687 community residents from Germany aged 65 years and older between May and August 2019, it was noted that physical and mental components of health-related quality of life were associated with socioeconomic, sociodemographic, psychosocial, and behavioural factors in adults aged 65 years and older [60]. In a cross-sectional survey across three urban centers in economically developed China on community-dwelling older adults, conducted between September and December 2021 on a sample of 1218 questionnaires, it was reported that different disease types have different degrees of impact on health-related quality of life, and that patients with multimorbidity have lower health-related quality of life [61].

These findings clearly indicate the complex influence of different domains and disease types on quality of life in older adults, thus proposing and implementing patient-oriented as well as specific measures in promoting positive lifestyle patterns and multidisciplinary, interdisciplinary, and transdisciplinary interventions is of great importance for improvement of overall functioning and health perspective outcomes along with quality of life. In addition, the promotion of older adults’ social participation is vital in terms of quality of life improvement.

## 9. Psychosocial Interventions

In an ageing society, the importance of preserving the psychosocial functioning is as important as the preservation and improvement of physical functioning. Moreover, health-related changes, demographic changes, and healthy ageing frameworks additionally point to the importance and necessity of the inclusion and implementation in caring models of older adults. In line with this, rather than putting the focus on disease prevention only, promotion of wellbeing in older adults is needed as well [62]. However, there are still challenges in terms of high-quality evidence studies performed, intervention methods organisation, and the unclear effects of interventional studies on the wellbeing of older adults [62].

Previous reports have presented the role and importance of psychological interventions in older adults across numerous domains. When performing therapy in older adults, for it to be effective, age-related adaptations should be considered, including the presence of comorbidities, cognitive capacity, as well as the role of family members or caregivers [63]. Moreover, further optimisation of treatment outcomes can be influenced by modifying treatment goals, such as increasing self-reliance, improving social or family functioning, reducing primary care service needs, as well as planning for long-term healthcare [63].

A systematic review and meta-analysis by Forsman et al., published in 2011, revealed that the effect of psychosocial interventions in reducing depressive symptoms in older adults is small but significant [64]. Additionally, a systematic review of randomized controlled trials by Pu et al., published in 2019, suggested that psychosocial interventions could be a potential alternative for pain management in older adults with dementia [65]. Furthermore, in a scoping review by De Lucia et al., published in 2024, the authors pointed out that eHealth multimodal interventions, including physical and psychosocial components in older adults with chronic non-cancer pain, have shown signs of effectiveness regarding targeted biopsychosocial outcomes, with overall positive participant engagement and satisfaction ratings [66]. It was previously stated that several psychosocial interventions for pain have been evaluated in older adults, including cognitive behavioural therapy, emotional disclosure, and mind–body interventions [67].

Cognitive behavioural therapy (CBT) is effective for depression, stress, anxiety, and chronic pain as well as in improving activities of daily living and quality of life [68]. In a systematic review and meta-analysis by Liu et al., published in 2018, it was suggested that such an intervention has effects on reducing the fear of falling and improving balance in older adults [69]. The advantage of cognitive behaviour therapy is that it can be tailored to an individual’s needs and specific situation [70]. However, several barriers should be addressed in implementing cognitive behaviour therapy, including transportation, reduced mobility for older persons, and limited availability of skilled therapists [70].

A study conducted in China from 2015 to 2017 on 72 participants found that the path-oriented psychological self-help intervention (P-oPSI) had benefits in improving the mental health of empty-nest older adults [71].

In a systematic review and meta-analysis by Li et al., published in 2023, it was reported that there were short-term effects of psychological interventions focusing on social skills and negativity-elimination in alleviating the feeling of loneliness during the COVID-19 pandemic in older adults [72].

Moreover, problem-solving therapy in older adults led to a reduction in depressive symptoms and signs. It is assumed that improvement in problem-solving skills in older patients leads to better coping with current or future difficulties [73]. In addition, interpersonal psychotherapy has been shown to be effective in the treatment of depression in older patients [73].

**Figure 3:**
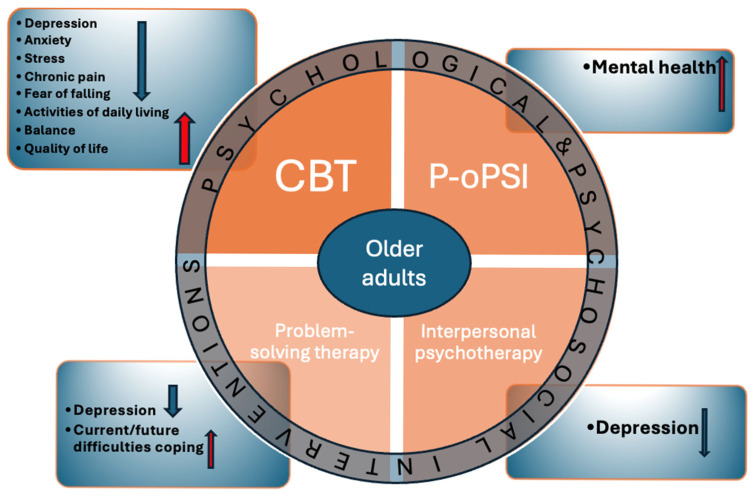
Psychological and psychosocial interventions in older adults and their effects.

In Figure 3 (original illustration created by the authors for this review), psychological and psychosocial interventions in older adults and their effects are presented.

## 10. The Role of Advanced Technology

Even though the technology is shown to have potential in the improvement of the quality of life and communication in older adults and their caregivers, the challenges still persist. In terms of ageing and technology research, it continues to be fragmented; there is a lack of a clear conceptual basis, quantitative health outcomes, and future directions [74].

Technology acceptance for the promotion of social participation in later life is a multicomplex process [75]. In a review on older adults and new technologies by Schroeder et al., it was reported that six domains, including demographic and health status, motivation, emotional awareness and needs, social influencers, knowledge and perception, as well as technology functional features, have an influence on the intention to use digital technology among older adults [76]. The importance of digital literacy as a significant determinant of quality of life in this population, mainly in the domains of social connectivity and health management, has previously been noted [77]. Moreover, there is a positive trend in internet use among adults aged 65 years and above, according to the Pew Research Center’s recent data [78]. Furthermore, shifts toward favourable technological adoptions were noticed during the pandemic, where the proportion of older adults who thought that internet use is essential increased, as well as the acceptance and uptake of technology for socialisation [78]. Additionally, older adults might benefit from technology in terms of providing them with rich cultural content as well as broader social interaction opportunities [79]. However, certain patterns in digital technology use are characteristic of older adults, such as smartphone preferences over other devices like computers and iPads, with a primary focus on communication and entertainment rather than advanced functions like online shopping and mobile payments [79].

A brief report by Siette et al., conducted in Australia within three data collecting periods (September 2018, March 2019, May 2020) on 21 older adults, stated that 80% and above of older adults used technology during the pandemic to maintain contact with family and friends, and there was no change in social networks [80]. In a cross-sectional survey study of 400 older adults from Canada during the COVID-19 pandemic by Haase et al., conducted in January 2021, further facilitators to using technology for socialization were identified as technology knowledge, accessibility of technology, reliance on others, and social motivation, while barriers included lack of interest and access as well as physical barriers [81].

Regarding the artificial intelligence (AI) technology and its application in older adults, in the scoping review by Ma et al., published in 2023, authors described five roles of AI technologies such as rehabilitation therapists, social facilitators, emotional supporters, supervisors and cognitive promoters as well as the fact that the AI technologies are capable of satisfying the unmet needs of older adults [82]. Advancements in machine learning/AI algorithms, user interface design, and sensor technology have resulted in better effectiveness and accessibility to older adults, with numerous benefits including early detection of health problems, improved medication adherence, extended independent living, reduced hospitalisations and improved quality of life [83]. However, challenges remain in terms of privacy use, technology adoption, and ease of use [83]. In the review by Lee and Allen, published in 2025, the authors reported that outcomes of AI interventions in older adults may include a reduction in loneliness and isolation as well as emotional expression and mental stimulation promotion [84].

In a study on older adults from rural areas of Western China, conducted between May and June 2024 on 311 respondents, it was found that a better understanding of smart health in older adults led the majority of participants to recognize its potential as a personal health management benefit, fulfilling daily needs, and contributing to rural development [85]. However, it is important to emphasise the presence of challenges in terms of barriers to technical use, economic limitations, and limitations regarding cognitive diversity in smart health [85].

Furthermore, in a scoping review by Budak et al., published in 2023, the authors suggested that ambient assisted living technologies showed a promising impact on mood, social engagement, and quality of life [86].

Additionally, in the review by Pratt et al., published in 2008, regarding psychosocial rehabilitation of older adults with serious mental illness, three new models of skills training were described: Functional Adaptation Skills Training (FAST), Cognitive-Behavioural Social Skills Training (CBSST), and Helping Older People Experience Success (HOPES), each of which had positive outcomes [87].

In Table 1, we presented the potential barriers to technology use and the digital divide in the older population.

Regarding the robots’ introduction into older adults’ daily tasks, it was stated that such technology can be considered as a potential support for older adults with mobility and cognitive impairments, particularly in terms of promoting independence, increasing abilities, establishing safety, and having a favourable impact on healthcare costs reduction [93]. Considering technological advances, the role of care robots in health and wellbeing support of older adults is promising, particularly in assisting in various domains from emotional support to physical activities and health management [94]. Furthermore, socially assistive robots (SAR) are designed to provide assistance and support as well as to promote positive and efficient interactions with human individuals, enhancing their quality of life via improvements in motivation, education, and rehabilitation [95].

Previous studies reported numerous benefits of ageing at home, including improved quality of life. In this regard, innovative technologies that will enable Smart Homes for the support of individuals to age at home are gaining interest. Functional as well as health-related benefits of Smart Homes were previously described [96]. In a systematic review and meta-analysis of Liu et al., published in 2019, it was stated that for older adults with chronic conditions, Smart Homes appear to have an effect on depression and physical functioning [97].

## 11. Conclusions, Challenges, and Future Considerations

A better understanding of the physiological, biological, psychological and social constructs of ageing along with improvement in technology and the availability of current knowledges and evidence-based studies on measures and interventions for improving the quality of life in older adults, could be beneficial in planning future directions for sensitive advancements between subtle and significant gaps in knowledge, practice and outcomes regarding new strategies, policymaking and healthcare interventions for the benefit of older adults.

It should be stated that older adults belong to a wide range of groups, including different educational levels, cognitive and psychological aspects, socio-economic status, and technological perceptions. These can present important challenges in addressing and providing optimal interventions in physical and psychosocial domains.

In line with this, promotion of self-care attitude and positive perception of ageing might be beneficial in improving the quality of life in older adults [46]. Furthermore, considering possible action for the reduction in social isolation and loneliness in older adults, pandemic age-friendly interventions, as well as strength-based approaches, are suggested for further exploration, such as outdoor activities, intergenerational programmes, and other outreach approaches as age-friendly interventions, and community and system-level capacity establishment as strength-based approaches [98]. Moreover, when considering technology implementation for social participation promotion in later life, an adapted/tailored training approach might potentially increase self-efficacy regarding technology use [75]. Overcoming the barriers for implementation of cognitive behaviour therapy in older adults is also a perspective strategy, including delivering the treatment over the telephone for chronic pain sufferers [70].

Moving beyond current knowledge and practice, in order to achieve better influence of proposed interventions and strategies in the older population and to achieve and maintain optimal quality of life multidimensionally, several proposals should be addressed, including but not limited to: personalization of new interventions, active involvement of older adults in interventions delivery and implementation, and integrative approaches towards psychosocial-health-digital interventions.

## Figures and Tables

**Table 1:** Potential barriers to technology use and the digital divide in the older population.

Barriers		References
Access and digital divide	Lack of/lower access, low technology literacy, lower usage of recent technologies Primary digital divide (Older adults without access) Secondary digital divide (Older adults with access, without using it)	[88,89,90]
Personal factors	Gender Willingness to learn and living arrangement Physical and cognitive challenges Privacy concerns, mistrust Education and income	[89,91]
**Factors affecting the digital divide**		
Access divide (AD)	Gender (for females is higher) Health (negative relationship with AD, poorer the health—higher AD)	[92]
Use divide (UD)	Gender (for females is higher) Health (negative relationship with UD, poorer the health—higher UD)	
Knowledge divide (KD)	Place of residency (higher KD in rural area) Educational level (higher the level—higher the KD)	

## Data Availability

No new data were created or analyzed in this study.

## References

[1] de Medeiros M.M.D., Carletti T.M., Magno M.B., Maia L.C., Cavalcanti Y.W., Rodrigues-Garcia R.C.M. (2020). Does the institutionalization influence elderly’s quality of life? A systematic review and meta-analysis. BMC Geriatr..

[2] GBD 2019 Ageing Collaborators (2022). Global, regional, and national burden of diseases and injuries for adults 70 years and older: Systematic analysis for the Global Burden of Disease 2019 Study. BMJ.

[3] World Health Organization Ageing and Health. https://www.who.int/news-room/fact-sheets/detail/ageing-and-health.

[4] Ferrucci L., Giallauria F., Guralnik J.M. (2008). Epidemiology of ageing. Radiol. Clin. N. Am..

[5] Asian Development Bank Adapting to Ageing Asia and the Pacific. https://www.adb.org/what-we-do/topics/social-development/aging-asia.

[6] Population Reference Bureau Fact Sheet: Ageing in the United States. http://www.prb.org/resources/fact-sheet-aging-in-the-united-states.

[7] United Nations Political Declaration and Madrid International Plan of Action on Ageing. Proceedings of the Second World Assembly on Ageing.

[8] Pruchno R. (2019). Technology and Ageing: An Evolving Partnership. Gerontologist.

[9] Czaja S.J. (2021). Current Findings and Issues in Technology and Aging. J. Appl. Gerontol..

[10] Marengoni A., Angleman S., Melis R., Mangialasche F., Karp A., Garmen A., Meinow B., Fratiglioni L. (2011). Ageing with multimorbidity: A systematic review of the literature. Ageing Res. Rev..

[11] Fabbri E., Zoli M., Gonzalez-Freire M., Salive M.E., Studenski S.A., Ferrucci L. (2015). Ageing and Multimorbidity: New Tasks, Priorities, and Frontiers for Integrated Gerontological and Clinical Research. J. Am. Med. Dir. Assoc..

[12] Drazen J.M., Fabbri L.M. (2014). Ageing and multimorbidity. Eur. Respir. J..

[13] Makovski T.T., Schmitz S., Zeegers M.P., Stranges S., van den Akker M. (2019). Multimorbidity and quality of life: Systematic literature review and meta-analysis. Ageing Res. Rev..

[14] Salive M.E. (2013). Multimorbidity in older adults. Epidemiol. Rev..

[15] Nicholson K., Liu W., Fitzpatrick D., Hardacre K.A., Roberts S., Salerno J., Stranges S., Fortin M., Mangin D. (2024). Prevalence of multimorbidity and polypharmacy among adults and older adults: A systematic review. Lancet Healthy Longev..

[16] Yen K.H., Hsu C.C., Yu P.C., Liu H.Y., Chen Z.J., Chen Y.W., Peng L.N., Lin M.H., Chen L.K. (2021). Determinants of improved quality of life among older adults with multimorbidity receiving integrated outpatient services: A hospital-based retrospective cohort study. Arch. Gerontol. Geriatr..

[17] Lo Y.T., Chen M.H., Lu T.H., Yang Y.P., Chang C.M., Yang Y.C. (2024). Effects of an integrated ambulatory care program on healthcare utilization and costs in older patients with multimorbidity: A propensity score-matched cohort study. BMC Geriatr..

[18] Xi J.Y., Liang B.H., Zhang W.J., Yan B., Dong H., Chen Y.Y., Lin X., Gu J., Hao Y.T. (2025). Effects of population ageing on quality of life and disease burden: A population-based study. Glob. Health Res. Policy.

[19] Rodrigues L.P., Vissoci J.R.N., França D.G., Caruzzo N.M., Batista S.R.R., de Oliveira C., Nunes B.P., Silveira E.A. (2022). Multimorbidity patterns and hospitalisation occurrence in adults and older adults aged 50 years or over. Sci. Rep..

[20] Rodrigues L.P., de Oliveira Rezende A.T., Delpino F.M., Mendonça C.R., Noll M., Nunes B.P., de Oliviera C., Silveira E.A. (2022). Association between multimorbidity and hospitalization in older adults: Systematic review and meta-analysis. Age Ageing.

[21] Zheng L.X., Walsh E.I., Sutarsa I.N. (2023). Provision of health services for elderly populations in rural and remote areas in Australia: A systematic scoping review. Aust. J. Rural Health.

[22] Luppa M., Luck T., Weyerer S., König H.H., Brähler E., Riedel-Heller S.G. (2010). Prediction of institutionalization in the elderly. A systematic review. Age Ageing.

[23] Fealy S., McLaren S., Nott M., Seaman C.E., Cash B., Rose L. (2024). Psychological interventions designed to reduce relocation stress for older people transitioning into permanent residential aged care: A systematic scoping review. Aging Ment. Health.

[24] Quaosar G.M.A.A., Hoque M.R., Bao Y. (2018). Investigating Factors Affecting Elderly’s Intention to Use m-Health Services: An Empirical Study. Telemed. J. E Health.

[25] Xie Z., Kalun O.C. Acceptance of mHealth by Elderly Adults: A Path Analysis. Proceedings of the Human Factors and Ergonomics Society Annual Meeting.

[26] Bambeni N. (2024). Perspective Chapter: Social Ageing Challenges Faced by Older Adults Exposed to Conditions of Underdevelopment and Extreme Poverty.

[27] Illario M., De Luca V., Onorato G., Tramontano G., Carriazo A.M., Roller-Wirnsberger R.E., Apostolo J., Eklund P., Goswami N., Iaccarino G. (2022). Interactions Between EIP on AHA Reference Sites and Action Groups to Foster Digital Innovation of Health and Care in European Regions. Clin. Interv. Aging.

[28] Dziechciaż M., Filip R. (2014). Biological psychological and social determinants of old age: Bio-psycho-social aspects of human aging. Ann. Agric. Environ. Med..

[29] Rutledge J., Oh H., Wyss-Coray T. (2022). Measuring biological age using omics data. Nat. Rev. Genet..

[30] Gellert P., Alonso-Perez E. (2024). Psychosocial and biological pathways to ageing: The role(s) of the behavioral and social sciences in geroscience. Z. Gerontol. Geriatr..

[31] Charles S.T., Carstensen L.L. (2010). Social and emotional aging. Annu. Rev. Psychol..

[32] Steptoe A., Deaton A., Stone A.A. (2015). Subjective wellbeing, health, and ageing. Lancet.

[33] Kang H., Kim H. (2022). Ageism and Psychological Wellbeing Among Older Adults: A Systematic Review. Gerontol. Geriatr. Med..

[34] Wernher I., Lipsky M.S. (2015). Psychological theories of aging. Dis. Mon..

[35] Levy B. (2009). Stereotype Embodiment: A Psychosocial Approach to Ageing. Curr. Dir. Psychol. Sci..

[36] World Health Organization Active Ageing: A Policy Framework. https://iris.who.int/bitstream/handle/10665/67215/WHO_NMH_NPH_02.8.pdf?sequence=1andisAllowe.

[37] Ayoubi-Mahani S., Eghbali-Babadi M., Farajzadegan Z., Keshvari M., Farokhzadian J. (2023). Active aging needs from the perspectives of older adults and geriatric experts: A qualitative study. Front. Public Health.

[38] Rowe J.W., Kahn R.L. (1997). Successful aging. Gerontologist.

[39] Meng X., D’Arcy C. (2014). Successful aging in Canada: Prevalence and predictors from a population-based sample of older adults. Gerontology.

[40] Jang S.N., Choi Y.J., Kim D.H. (2009). Association of socioeconomic status with successful ageing: Differences in the components of successful ageing. J. Biosoc. Sci..

[41] Harada C.N., Natelson Love M.C., Triebel K.L. (2013). Normal cognitive aging. Clin. Geriatr. Med..

[42] Depp C., Vahia I.V., Jeste D. (2010). Successful aging: Focus on cognitive and emotional health. Annu. Rev. Clin. Psychol..

[43] Mitina M., Young S., Zhavoronkov A. (2020). Psychological aging, depression, and wellbeing. Aging.

[44] Fernández-Ballbé Ó., Martin-Moratinos M., Saiz J., Gallardo-Peralta L., Barrón López de Roda A. (2023). The Relationship between Subjective Ageing and Cognition in Elderly People: A Systematic Review. Healthcare.

[45] Tully-Wilson C., Bojack R., Millear P.M., Stallman H.M., Allen A., Mason J. (2021). Self-perceptions of ageing: A systematic review of longitudinal studies. Psychol. Aging.

[46] Velaithan V., Tan M.M., Yu T.F., Liem A., The P.L., Su T.T. (2024). The Association of Self-Perception of Ageing and Quality of Life in Older Adults: A Systematic Review. Gerontologist.

[47] European Center for Social Welfare Policy and Research The End of “Active Ageing”. https://www.euro.centre.org/publications/detail/4727.

[48] Mitchell D.M., Henry A.J., Ager R.D. (2023). COVID-19 impacts and interventions for older adults: Implications for future disasters. J. Gerontol. Geriatr..

[49] Holt N.R., Neumann J.T., McNeil J.J., Cheng A.C. (2020). Implications of COVID-19 for an ageing population. Med. J. Aust..

[50] Mushtaq A., Khan M.A. (2024). Social isolation, loneliness, and mental health among older adults during COVID-19: A scoping review. J. Gerontol. Soc. Work.

[51] Briere J., Wang S.H., Khanam U.A., Lawson J., Goodridge D. (2023). Quality of life and wellbeing during the COVID-19 pandemic: Associations with loneliness and social isolation in a cross-sectional, online survey of 2,207 community-dwelling older Canadians. BMC Geriatr..

[52] Derrer-Merk E., Reyes-Rodriguez M.F., Soulsby L.K., Roper L., Bennett K.M. (2023). Older adults’ experiences during the COVID-19 pandemic: A qualitative systematic literature review. BMC Geriatr..

[53] Izekenova A., Izekenova A., Sukenova D., Nikolic D., Chen Y., Rakhmatullina A., Nurbakyt A. (2025). Factors Associated with Loneliness and Psychological Distress in Older Adults During the COVID-19 Pandemic in Kazakhstan: A Cross-Sectional Study. Medicina.

[54] Martins Van Jaarsveld G. (2020). The Effects of COVID-19 Among the Elderly Population: A Case for Closing the Digital Divide. Front. Psychiatry.

[55] Adhikari R., Shah R., Ghimire K., Khanal B., Baral S., Adhikari A., Malla D.K., Khanal V. (2025). The Quality of Life and Associated Factors Among Older Adults in Central Nepal: A Cross-Sectional Study Using the WHOQOL-OLD Tool. Int. J. Environ. Res. Public Health.

[56] Marzo R.R., Khanal P., Shrestha S., Mohan D., Myint P.K., Su T.T. (2023). Determinants of active aging and quality of life among older adults: Systematic review. Front. Public Health.

[57] Çiftci N., Yıldız M., Yıldırım Ö. (2023). The effect of health literacy and health empowerment on quality of life in the elderly. Psychogeriatrics.

[58] World Health Organization Healthy Ageing and Functional Ability. https://www.who.int/news-room/questions-and-answers/item/healthy-ageing-and-functional-ability.

[59] Siqeca F., Yip O., Mendieta M.J., Schwenkglenks M., Zeller A., De Geest S., Zúñiga F., Stenz S., Briel M., Quinto C. (2022). Factors associated with health-related quality of life among home-dwelling older adults aged 75 or older in Switzerland: A cross-sectional study. Health Qual. Life Outcomes.

[60] Geigl C., Loss J., Leitzmann M., Janssen C. (2023). Social factors of health-related quality of life in older adults: A multivariable analysis. Qual. Life Res..

[61] Liang X., Wei H., Mo H., Yang G., Wan L., Dong H., He Y. (2024). Impacts of chronic diseases and multimorbidity on health-related quality of life among community-dwelling elderly individuals in economically developed China: Evidence from cross-sectional survey across three urban centers. Health Qual. Life Outcomes.

[62] Iwano S., Kambara K., Aoki S. (2022). Psychological Interventions for Wellbeing in Healthy Older Adults: Systematic Review and Meta-Analysis. J. Happiness Stud..

[63] Kennedy G.J., Tanenbaum S. (2000). Psychotherapy with older adults. Am. J. Psychother..

[64] Forsman A.K., Schierenbeck I., Wahlbeck K. (2011). Psychosocial interventions for the prevention of depression in older adults: Systematic review and meta-analysis. J. Aging Health.

[65] Pu L., Moyle W., Jones C., Todorovic M. (2019). Psychosocial interventions for pain management in older adults with dementia: A systematic review of randomized controlled trials. J. Adv. Nurs..

[66] De Lucia A., Perlini C., Chiarotto A., Pachera S., Pasini I., Del Piccolo L., Donisi V. (2024). eHealth-Integrated Psychosocial and Physical Interventions for Chronic Pain in Older Adults: Scoping Review. J. Med. Internet Res..

[67] Keefe F.J., Porter L., Somers T., Shelby R., Wren A.V. (2013). Psychosocial interventions for manageing pain in older adults: Outcomes and clinical implications. Br. J. Anaesth..

[68] Lim J.A., Choi S.H., Lee W.J., Jang J.H., Moon J.Y., Kim Y.C., Kang D.H. (2018). Cognitive-behavioral therapy for patients with chronic pain: Implications of gender differences in empathy. Medicine.

[69] Liu T.W., Ng G.Y.F., Chung R.C.K., Ng S.S.M. (2018). Cognitive behavioural therapy for fear of falling and balance among older people: A systematic review and meta-analysis. Age Ageing.

[70] Wilkinson P. (2013). Cognitive behavioural therapy with older people. Maturitas.

[71] Wang L.N., Tao H., Wang M., Yu H.W., Su H., Wu B. (2019). Efficacy of path-oriented psychological self-help interventions to improve mental health of empty-nest older adults in the Community of China. BMC Psychiatry.

[72] Li M., Rao W., Su Y., Sul Y., Caron G., D’Arcy C., Fleury M.J., Meng X. (2023). Psychological interventions for loneliness and social isolation among older adults during medical pandemics: A systematic review and meta-analysis. Age Ageing.

[73] Klausner E.J., Alexopoulos G.S. (1999). The future of psychosocial treatments for elderly patients. Psychiatr. Serv..

[74] Pilotto A., Boi R., Petermans J. (2018). Technology in geriatrics. Age Ageing.

[75] Benoit-Dubé L., Jean E.K., Aguilar M.A., Zuniga A.M., Bier N., Couture M., Lussier M., Lajoie X., Belchior P. (2023). What facilitates the acceptance of technology to promote social participation in later life? A systematic review. Disabil. Rehabil. Assist. Technol..

[76] Schroeder T., Dodds L., Georgiou A., Gewald H., Siette J. (2023). Older Adults and New Technology: Mapping Review of the Factors Associated With Older Adults’ Intention to Adopt Digital Technologies. JMIR Aging.

[77] Xin Y., Weina H., Yan D. (2025). Digital literacy impacts quality of life among older adults through hierarchical mediating mechanisms. Sci. Rep..

[78] Czaja S.J., Charness N., Rogers W.A., Sharit J., Moxley J.H., Boot W.R. (2024). The Benefits of Technology for Engaging Aging Adults: Findings From the PRISM 2.0 Trial. Innov. Aging.

[79] Li S., Yunus M.M., Hussain R.B.B.M., Lin W. (2025). Cultural adaptation to aging: A study on digital cultural adaptation needs of Chinese older adults based on KANO model. Front. Public Health.

[80] Siette J., Dodds L., Seaman K., Wuthrich V., Johnco C., Earl J., Dawes P., Westbrook J.I. (2021). The impact of COVID-19 on the quality of life of older adults receiving community-based aged care. Australas. J. Ageing.

[81] Haase K.R., Cosco T., Kervin L., Riadi I., O’Connell M.E. (2021). Older Adults’ Experiences with Using Technology for Socialization During the COVID-19 Pandemic: Cross-sectional Survey Study. JMIR Aging.

[82] Ma B., Yang J., Wong F.K.Y., Wong A.K.C., Ma T., Meng J., Zhao Y., Wang Y., Lu Q. (2023). Artificial intelligence in elderly healthcare: A scoping review. Ageing Res. Rev..

[83] McDaniel L., Essien I., Lefcourt S., Zelleke E., Sinha A., Chellappa R., Abadir P.M. (2025). Aging With Artificial Intelligence: How Technology Enhances Older Adults’ Health and Independence. J. Gerontol. A Biol. Sci. Med. Sci..

[84] Lee J., Allen J. (2025). Bridging the Gap: The Role of AI in Enhancing Psychological Wellbeing Among Older Adults. Psychol. Int..

[85] Li X.Y., Li J., Zhu N.L., Luo L., Zhang S.Y., Cheng W.W., Li J.X., Yu C., Lu S.H., Zhu L. (2025). When “Aging” meets “Intelligence”: Smart health cognition and intentions of older adults in rural Western China. Front. Psychiatry.

[86] Budak K.B., Atefi G., Hoel V., Uribe F.L., Meiland F., Teupen S., Felding S.A., Roes M. (2023). Can technology impact loneliness in dementia? A scoping review on the role of assistive technologies in delivering psychosocial interventions in long-term care. Disabil. Rehabil. Assist. Technol..

[87] Pratt S.I., Van Citters A.D., Mueser K.T., Bartels S.J. (2008). Psychosocial rehabilitation on older adults with serious mental illness: A review of the research literature and recommendations for development of rehabilitative approaches. Am. J. Psychiatr. Rehabil..

[88] Leff B., Ritchie C.S., Rising K.L., Cannon K., Wardlow L. (2025). Addressing barriers to equitable telehealth for older adults. Front. Med..

[89] Zhang M. (2023). Older people’s attitudes towards emerging technologies: A systematic literature review. Public Underst. Sci..

[90] Wu Y.H., Damnée S., Kerhervé H., Ware C., Rigaud A.S. (2015). Bridging the digital divide in older adults: A study from an initiative to inform older adults about new technologies. Clin. Interv. Aging.

[91] Hepburn J., Williams L., McCann L. (2025). Barriers to and Facilitators of Digital Health Technology Adoption Among Older Adults With Chronic Diseases: Updated Systematic Review. JMIR Aging.

[92] Zhang K., Cheng X., Li D., Meng X. (2025). The Digital Divide of Older People in Communities: Urban-Rural, Gender, and Health Disparities and Inequities. Health Soc. Care Community.

[93] Olatunji S.A., Shim J.S., Syed A., Tsai Y.L., Pereira A.E., Mahajan H.P., Mudar R.A., Rogers W.A. (2025). Robotic support for older adults with cognitive and mobility impairments. Front. Robot. AI.

[94] Lee S.H., Kim J.S., Yu S. (2025). The impact of care robots on older adults: A systematic review. Geriatr. Nurs..

[95] Zguda P., Radosz-Knawa Z., Kukier T., Radosz M., Kamińska A., Indurkhya B. (2025). How Do Older Adults Perceive Technology and Robots? A Participatory Study in a Care Center in Poland. Electronics.

[96] Aggar C., Sorwar G., Seton C., Penman O., Ward A. (2023). Smart home technology to support older people’s quality of life: A longitudinal pilot study. Int. J. Older People Nurs..

[97] Liu P., Li G., Jiang S., Liu Y., Leng M., Zhao J., Wang S., Meng X., Shang B., Chen L. (2019). The effect of smart homes on older adults with chronic conditions: A systematic review and meta-analysis. Geriatr. Nurs..

[98] Kadowaki L., Wister A. (2023). Older Adults and Social Isolation and Loneliness During the COVID-19 Pandemic: An Integrated Review of Patterns, Effects, and Interventions. Can. J. Aging.

